# Identifying the synaptic origin of ongoing neuronal oscillations through spatial discrimination of electric fields

**DOI:** 10.3389/fncom.2013.00005

**Published:** 2013-02-12

**Authors:** Antonio Fernández-Ruiz, Oscar Herreras

**Affiliations:** Experimental and Computational Neurophysiology, Department of Systems Neuroscience, Cajal Institute - Consejo Superior de Investigaciones CientíficasMadrid, Spain

**Keywords:** local field potentials, gamma oscillations, spatial discrimination, independent component analysis, spontaneous activity

## Abstract

Although intracerebral field potential oscillations are commonly used to study information processing during cognition and behavior, the cellular and network processes underlying such events remain unclear. The limited spatial resolution of standard single-point recordings does not clarify whether field oscillations reflect the activity of one or many afferent presynaptic populations. However, multi-site recording devices now provide high-resolution spatial profiles of local field potentials (LFPs) and when coupled to modern mathematical analyses that discriminate signals with distinct but overlapping spatial distributions, they open the door to better understand these potentials. Here we review recent insights that help disentangle certain pathway-specific activities. Accordingly, some oscillatory patterns can now be viewed as a periodic succession of synchronous synaptic currents that reflect the time envelope of spiking activity in given presynaptic populations. These analyses modify our concept of brain rhythms as abstract entities, molding them into mechanistic representations of network activity and allowing us to work in the time domain, reducing the loss of information inherent to data-chopping frequency treatment.

## Field potential oscillations: how much do we know?

The incessant processing of information by neural circuits has been investigated using a variety of techniques, of which only a few have sufficient spatial and temporal resolution to grasp the rapid changes in electrical activity associated with brain function. Some record global activity in a non-invasive manner, such as EEG or magnetoencephalograms (MEG), while intracerebral approaches capture more local activity in particular regions, like local field potentials (LFPs). In all cases, one can appreciate the conspicuous presence of repetitive wave-like patterns or oscillations. Despite constituting a small fraction of the total brain activity, oscillations have received much attention as they can be readily associated to cognitive and behavioral tasks. The question is: how much do we know about the identity of the neuron populations involved in their generation?

Brain oscillations appeared well-suited for application of spectral analysis in order to quantify temporal patterns (Fourier analysis and its derivatives; Jenkins and Watts, 1968; Oppenheim and Schafer, 1989). Bioelectrical signals are usually filtered in predefined frequency bands of interest, a process that, it must be remembered, leads to the loss of a considerable amount of information. Such a jump from the time to the frequency domain summarizes temporal information and makes it handy to associate and compare with other measurements of a brain at work. Some paradigmatic cases are the identification of phases in the sleep cycle by their spectral content (Dement and Kleitman, 1957), the predictive character of hippocampal theta in terms of behavioral performance (Buzsáki et al., 1983), or the processing of visual stimuli contingent with cortical gamma activity (Gray et al., 1989).

Importantly, whether oscillatory or irregular, LFPs are complex signals that vary not only in time but also in space, as they are raised by uneven summation of currents originated in different sites, possibly even different oscillatory generators. Despite the practical advantages, frequency bands are not true physiological signals. All too frequently they are thought of and handled as if they were independent from each other, under a reductionist assumption that each constitutes a separate physiological entity. An increasing number of reports are now appearing in the literature challenging, questioning or simply describing inconsistent frequency bands in different or even the same brain areas (Florian et al., 1998; Csicsvari et al., 2003; Schmidt et al., 2009; Ray and Maunsell, 2011). One notable example is the open dispute regarding the physiological role of gamma activity (e.g., Nunez and Srinivasan, 2010) as a temporal reference frame bringing together different features of a visual stimulus (Singer and Gray, 1995; Fries et al., 2007). Recent results challenge this view, arguing that visual stimuli generate gamma activity at different frequencies in sub-regions of the visual cortex (Ray and Maunsell, 2010). Others even questioned their very existence, alleging that the spectral properties of the activity recorded are indistinguishable from filtered noise (Burns and Xing, 2011).

We are moving away from the old view of frequency bands as behavioral or cognitive flags. We now admit that LFP oscillations are highly variable over time and they have a flexible spectrum (Rivas et al., 1996; Bullock et al., 2003; Ray and Maunsell, 2010). However, it is uncertain what this means in terms of the afferent and target populations. For instance, oscillatory 40–50 Hz patterns in the visual cortex (Gray et al., 1989) or the hippocampus (Fernández-Ruiz et al., 2012a) are probably unrelated phenomena with different cellular mechanisms and having distinct computational meaning within their respective networks. On the other hand, the activity of a population of neurons undergoes variable frequency modulation, even during the same behavioral state (Reich et al., 1997; Czurkó et al., 1999; Chang et al., 2012) and hence, the temporal structure of the synaptic currents they originate in target neurons would be expected to change accordingly.

Indeed, when changes of spectral power of LFPs are interpreted, it becomes evident that there is insufficient knowledge on the scaling of unitary to macroscopic activities. The problem is clear for recordings obtained with non-invasive techniques due to the inherent difficulties in identifying deep generators (Gloor, 1985; Baillet et al., 2001; Srinivasan et al., 2006). Even when recording at the physical location of the generating sources, there is significant uncertainty. We can emphasize the dimension of the problem by considering a non-exhaustive list of possible causes that could lead to increased gamma power: (1) increased gamma-modulated excitation, (2) inhibition (3), or both; (4) the enhanced driving force of an unchanged rhythmic input by sustained changes in another input to the same neurons; (5) variations in phase-locking of presynaptic neurons or (6) in the number of units recruited to firing; (7) reduction of a concomitant antiphase rhythm near the recording electrode; (8) the powering of a different in-phase generator or (9) the addition of new ones; (10) variation in resonant intrinsic currents, and so on. The possibilities are many, some of a unitary origin and others network based.

Since the signals from multiple generators overlap spatially, customary measurements of frequency, phase and amplitude may be due to changes in a local or a distant source. These issues stress the importance of identifying the population/s contributing to a given field potential oscillation as a necessary step to infer on its physiological and computational meaning.

## Finding the current sources for LFPs: insights from spatially discriminating techniques

As indicated above, the problem of identifying the cellular origin of field oscillations, or the *inverse problem* as it has become known, is a fundamental issue in Systems Neuroscience. In simple terms, given an experimental macroscopic signal the amplitude of which varies at different sites (e.g., LFP, EEG, MEG), how can the location and extension of the generating source be determined? To put this in more applicable terms, can we reconstruct the characteristics of the electric currents generated by myriads of scattered microscopic sources co-activated as one, if their number (hence their position) change constantly? We know from theory that multiple combinations of independent sources (groups of active neurons) may give rise to a recorded signal with the same spatial pattern. There is no unique solution and in most cases, it is difficult or impossible to confirm the potential solutions experimentally. One would think the way out might be easier for LFPs that can be recorded at the site. However, whereas remote recordings lack necessary detail of the location and extension of current sources, the local recordings can't discriminate between local and remote (volume propagated) contributions.

A common feature of electric fields in the brain is that they vary spatially in a complex manner, on account of the shifting activation of neuron generators with irregular morphology and distribution. It is beyond the scope of this minireview to consider all the factors that are relevant to LFPs (see Elul, 1972 for a starting reading) and thus, we will focus on a few that are important for the discrimination of mixed LFP generators. Neurons with dominant axial geometry act as strong current dipoles (Lorente de No, 1947) and as such are the main contributors to field potentials. LFPs are contributed mostly by synaptic currents that, contrary to propagating spike currents, remain at the site of the synapse for their largest part (Varona et al., 2000; López-Aguado et al., 2002). It is intuitive that a common afferent input to one or another subcellular domain of the neuron population will give rise to different field potential profiles (Makarova et al., 2011). This fact underlies the laminar distribution of pathway-specific LFPs in regular structures (Korovaichuk et al., 2010). The problem arises when several pathways are co-activated, as is usually the case. In such circumstances the electric currents mix unevenly at different sites, and field potential gradients become complex and variable. That is why stable oscillations with seemingly identical waves, one after another, appeared advantageous as they are more accessible to repeated experimental screening. However, they may still be contributed by several inputs with varying magnitude (Makarov et al., 2010; Fernández-Ruiz et al., 2012a). Thus, only high-density recordings simultaneously performed at several depths can correctly map for spatial variations in successive waves originated by modulations in one or more of the contributing sources.

**Figure 1:**
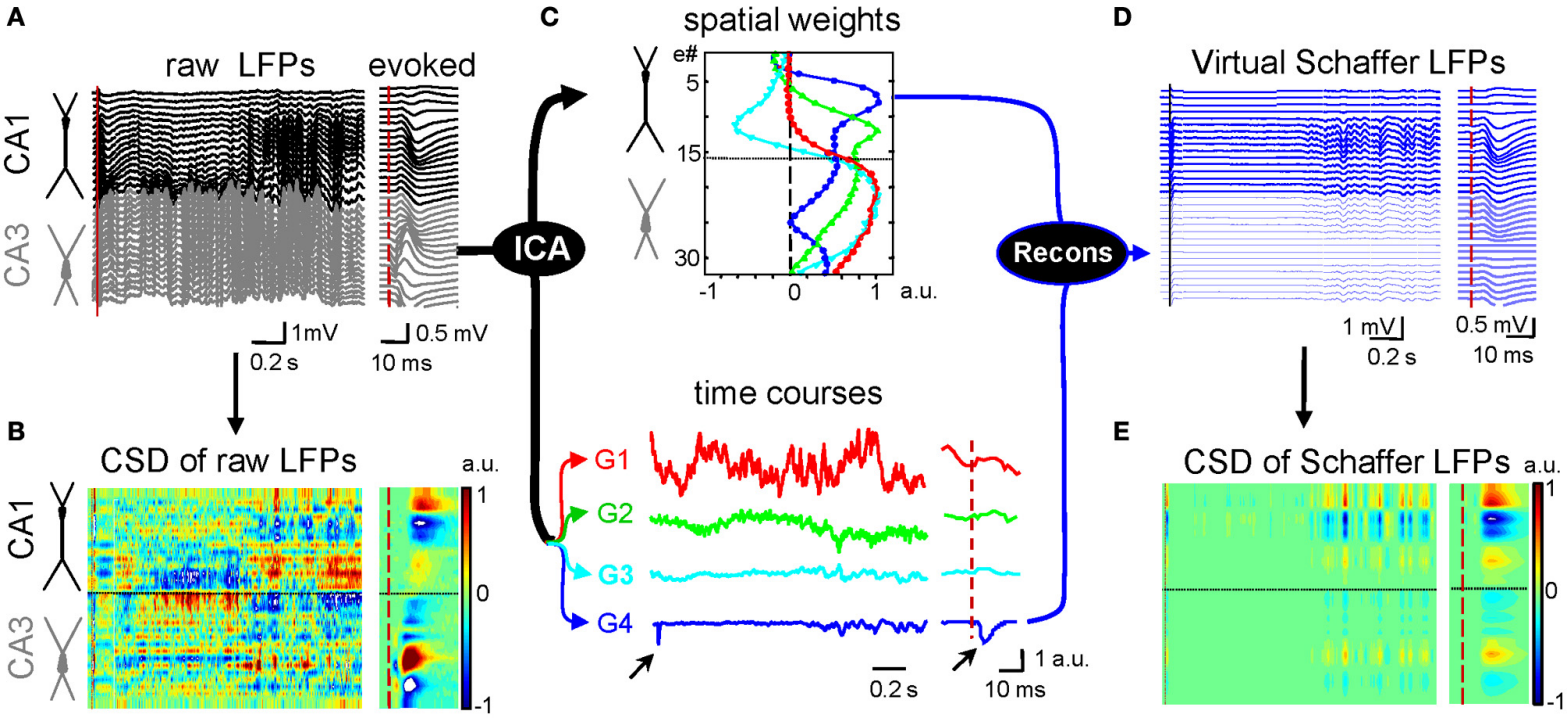
**Application of ICA to disentangle pathway-specific hippocampal LFPs. (A)** Ongoing raw LFPs across the CA1 and CA3 fields (black and gray traces, respectively). The dashed red line marks the time of a subthreshold stimulus applied to the ipsilateral CA3. The evoked field potential is amplified in the right inset. **(B)** CSD of the evoked potential (right) yields the standard distribution of inward (blue) and outward (yellow-red) currents across the CA1 region, while that of ongoing LFPs (left) renders a complex poorly informative mixture. **(C)** ICA of LFPs provides four main LFP generators, each defined by the curve of spatial weights (top panel) and a time course (bottom traces). Note that only the Schaffer generator (G4) captures the Schaffer-evoked activity (arrows). **(D)** Reconstructed (virtual) Schaffer LFPs for the raw LFP segment and evoked potential analyzed. The pronounced activity at electrodes 5–10 in the second half of the segment corresponds to a complex of sharp waves. **(E)** CSD of the virtual Schaffer LFPs provides precise spatiotemporal maps of inward/outward currents for unique spatially coherent membrane events. Note how clean the map of currents is after the concomitant activity elicited by other inputs is eliminated. (Modified from Fernández-Ruiz et al., 2012a).

Multisite linear recordings are well-suited to a method that has been employed to find the current generators underlying field potentials, known as current source density (CSD) analysis (Freeman and Nicholson, 1975). This approach has been very useful to determine the contributing cells and the location of synaptic membranes activated by afferent stimuli in laminar structures, such as the hippocampus or neocortex (Leung, 1979; Herreras, 1990; Schroeder et al., 1998). However, while interpreting CSD maps is simple for voltage profiles elicited by stimulating only one afferent pathway (Figures 1A,B, right panels), their application to ongoing LFPs renders complex spatial maps of intermingled inward and outward currents (left panels), and in general it is not feasible to identify the multiple synaptic generators. Partial success has been obtained in a few stereotypic LFP patterns, such as sharp-waves (SPWs: Ylinen et al., 1995), or the theta (Brankačk et al., 1993) and gamma rhythms (Csicsvari et al., 2003) in the hippocampus. But not even in these cases has it been possible to unequivocally determine whether one or several inputs contribute to the field oscillation due to unavoidable technical artifacts.

Amongst the approaches used to address the mixed contribution of inputs to macroscopic patterns, some sought the selective manipulation of parts of a network, such as the activation or the silencing of specific pathways or neuron types through electrical, optogenetic, or pharmacological intervention (Wu et al., 1998; Happel et al., 2010; Kuki et al., 2012). Other approaches pursued the disentanglement of LFPs into their original generators by applying statistical tools and algorithms (Di et al., 1990; Kocsis et al., 1999; Montgomery et al., 2009; Makarov et al., 2010). Blind source separation techniques, like the independent component analysis (ICA: Comon, 1994; Choi et al., 2005), appear to be the best suited by their capacity to find stable groups of sensors picking up a signal whose origin is stationary in the space, a feature that can be assumed for electrical fields generated by synaptic currents.

## Independent component analysis as a tool to reveal the cellular generators of brain oscillations

ICA is employed routinely in scalp EEG, MEG, and fMRI to find spatially stable patterns of coherent activity (Makeig et al., 1997; Tang et al., 2002; Kalcher et al., 2012). Recordings distant from the sources are highly sensitive to a variety of distortions in the path of the electrical currents (López-Aguado et al., 2001). Thus, they are limited to a coarse localization of large electrical sources of unknown cellular origin. The disturbing factors are less relevant in intracerebral recordings where electrodes can be placed directly on the generating sources, thereby minimizing the path of the currents to the recording electrode. The ICA operates on multiple simultaneously recorded signals and discriminates the contributing sources on the basis of their distinct spatial distribution. It is important to note that these components may be temporally correlated (Bell and Sejnowski, 1995; Makarova et al., 2011).

We have developed an implementation based on the ICA to separate the different synaptic pathways converging on hippocampal neurons on the evidence that each produces field potentials of stable and distinct spatial distribution (Makarov et al., 2010). The procedure is illustrated in Figure 1, in which each LFP component or generator is defined by two elements: a curve of spatial weights and the time course of the activity over the period analyzed (Figure 1C). We found that only a few macroscopic pathway-specific generators account for most of the LFP variance. Some are easy to identify by the characteristic profile of spatial weights, such as the excitatory Schaffer input from CA3 to CA1 that match the customary profiles of Schaffer-evoked fEPSPs (blue trace). This LFP generator exclusively captures all the activity elicited by this pathway, whether irregular or oscillatory, ongoing (SPWs) or stimulus evoked (Korovaichuk et al., 2010). Once disentangled from native LFPs, a given generator can be reconstructed in isolation and the virtual pathway-specific LFPs subjected to CSD analysis (Figures 1D,E).

## A mechanistic use for neural oscillations

**Figure 2:**
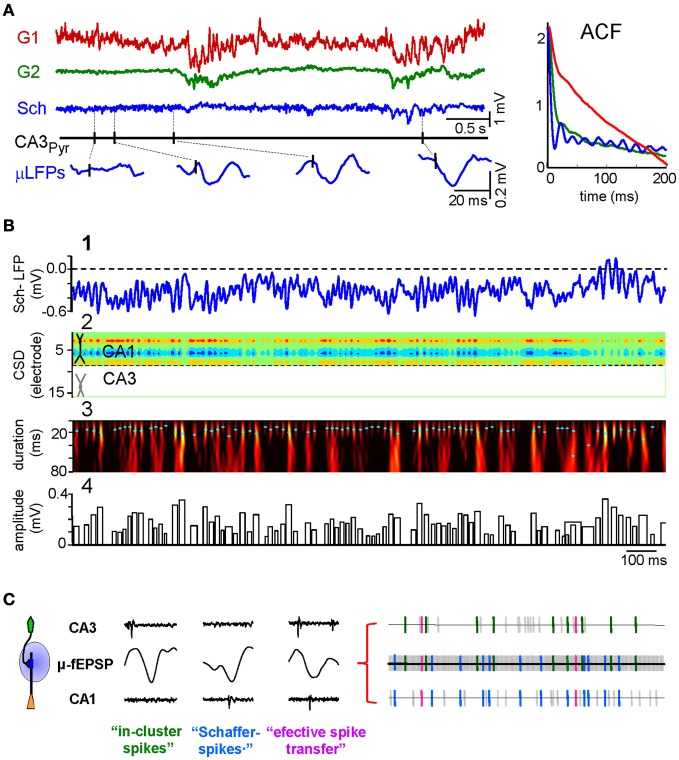
**CA3 to CA1 gamma input is a succession of elementary μ-fEPSPs that link pre- and postsynaptic units. (A)** Representative example of time courses of LFP generators and firing of a CA3 pyramidal cell. The baseline activity of the Schaffer LFP generator (in blue) is formed by a temporal succession of small wavelets or μ-fEPSP (enlargements at the bottom) in a global gamma pattern exclusive for this input. The presence of occasional sharp-waves (SPWs) is highlighted (in cyan). Autocorrelations (ACF) of the time courses of the generators are shown in the right inset. **(B)** (1) Fragment of Schaffer-LFP. Note the striking non-overlapping succession of wavelets. (2) The CSD analysis reveals a succession of currents with a spatial distribution matching that of Schaffer evoked potentials and SPWs. (3) The Schaffer-LFP in the wavelet domain. High magnitude (color coded from black to yellow) at given time instant and scale (cyan dots mark maxima) corresponds to the presence of μ-fEPSPs. (4) The width and height of the bar codify the duration and amplitude of detected μ-fEPSPs, respectively. **(C)** Using the excitatory quanta composing the baseline activity of Schaffer-LFPs (μ-fEPSPs) allows discriminating synaptically connected CA3 and CA1 units. The illustration of point processes in the left represents (from top to bottom) the spike train of a presynaptic CA3 pyramidal cell, the temporal series of μ-fEPSP events and a spike train of a postsynaptic CA1 pyramidal cell. Plausible monosynaptic coincidences are color coded as follows: Type I, green (in-cluster spikes); Type II, blue (Schaffer spikes); Type III, magenta (efficient spike transfer). Examples of these correlations are shown in right insets. (**A** and **B**) Modified from Fernández-Ruiz et al. (2012a); **(C)** modified from Fernández-Ruiz et al. (2012b).

Spike activity of a given presynaptic population elicits postsynaptic currents in target regions that if spatially appropriate, may set rhythmic, irregular, and more commonly, behaviorally modulated periods of different LFP patterns. As a case in point, the CA3 input to CA1 pyramidal cells is responsible for (1) low amplitude steady or (2) theta modulated gamma activity, and (3) isolated hyper-synchronous SPWs (Ylinen et al., 1995; Penttonen et al., 1998; Fernández-Ruiz et al., 2012a). The separation of the Schaffer activity from concomitant inputs allowed a detailed study of its time course. A small LFP epoch is presented in Figure 2 to show the independent activity of three different LFP generators. One is very irregular, while another presents high amplitude slow-waves and the third corresponds to the ongoing Schaffer input. In anesthetized animals, whereas epochs of gamma activity can be found in all LFP generators (Makarov et al., 2010) it only has a steady presence in the Schaffer input. Traditionally, gamma activity in the hippocampus was assumed to be mainly inhibitory (Mann and Paulsen, 2007; Buzsáki and Wang, 2012), in compliance with the observations of interneurons firing at that rate. But in phase firing of units to LFPs does not establish cause or effect, as the LFPs may reflect compound synaptic currents generated by spiking in unrecorded distant neurons. In fact, our findings refuted that view, as the gamma activity in the CA1 Schaffer generator is made up of small wave-like LFP events (~120 μV, ~16 ms long) that were specifically time locked with monosynaptic latency to the spikes of CA3 pyramidal cells but not interneurons. Indeed, the CSD analysis of the disentangled Schaffer-specific LFPs returns gamma sequence of current sinks in the CA1 stratum radiatum with a spatial distribution that tightly matched that of SPW events and CA3-evoked fEPSPs (Figure 2B2). These wavelets were blocked by pharmacologically silencing the CA3 or blockade of glutamate receptors in the CA1. Thus, the gamma rhythm recorded in this stratum of the CA1 region is a periodic succession of micro (μ)-fEPSPs triggered in CA1 pyramidal cells by CA3 assemblies firing in gamma sequence. This is probably the clearest demonstration of the cellular nature of a field oscillation in the intact brain presented to date.

The demonstration that Schaffer-LFPs are succession of quantal excitatory packages elicited by synchronous firing of functional assemblies of presynaptic units opens several possibilities. For instance, one may identify pairs of monosynaptically connected CA3 and CA1 neurons, enabling the ongoing spike transfer between pairs of cells of different populations and their plastic modulations to be studied *in vivo* (Fernández-Ruiz et al., 2012a,b). This was done by correlating pre- (CA3) and postsynaptic (CA1) spikes with the time series of Schaffer μ-fEPSPs. Also, several CA3 units fire synchronously and time-locked to Schaffer μ-fEPSPs in CA1, thus forming a functional assembly. We found that CA3 assemblies are not rigid constructs, as the neurons that comprise them may belong to different clusters and they do not always fire together, rather just a proportion of them (ca. 20%). In the CA1, we found that all pyramidal cells fired a fraction of their spikes time locked to Schaffer μ-fEPSPs. We term these as Schaffer-spikes, emphasizing the origin of the synaptic input that triggers them. The sorting of CA1 spikes by their synaptic drive opens the possibility to investigate the influence of different inputs to the elaboration of the output code of individual neurons. We also found triple coincidences, i.e., a presynaptic CA3 spike elicits a μ-fEPSP that in turn fires a postsynaptic spike. We call this as an effective spike transfer between two nodes in a network. Thus, pairs of pre- and postsynaptic spikes can be selected by their time-locking to a common μ-fEPSP, revealing temporal correlations that would normally remain buried in standard dual correlations of spike trains.

Another practical example of the use of pathway-specific LFPs is to study ongoing correlates of plastic phenomena. Thus, following induction of LTP by CA3 stimulation we found a moderate increase in the gamma power of raw LFPs in the CA1, but a large specific increase in the power of the ICA-separated Schaffer generator (while other components of the LFP remained unchanged: Fernández-Ruiz et al., 2012b). Hence, the ongoing modulations of a given synaptic pathway may not be discernible in raw mixed LFPs, whereas they can be observed, quantified and compared with other activities after their separation into pathway-specific LFP generators.

## Concluding remarks

By studying the wide band time course of ICA-isolated LFP generators we gain much more information on network dynamics than by chopping LFPs into narrow frequency bands and analyzing them separately. Hence, we consider that the entity with true physiological meaning coming out of LFPs is the LFP generator, rather than a particular temporal pattern in native LFPs that may still be a composite signal.

The example used here of the CA3 gamma input to CA1 may be generalized to other oscillatory and irregular patterns. The use of pathway-specific LFPs eliminates important concern as to the possibility of an LFP oscillation being a mixture of two or more inputs, and allows a direct interpretation of their fluctuations as time envelopes of synaptic currents with a precise identification of the populations of origin and destination. Thus, we may be close to determining which oscillations are periodic successions of excitatory or inhibitory postsynaptic potentials. The reconstruction of pre- and postsynaptic time sequences represents a step toward a mechanistic understanding of information transfer in identified segments of a network. For these reasons, it may be the moment to recover a feeling for the temporal and spatial domains, so as to complement and extend the spectral characterization of oscillatory patterns.

### Conflict of interest statement

The authors declare that the research was conducted in the absence of any commercial or financial relationships that could be construed as a potential conflict of interest.

## References

[B1] Baillet S. Mosher J. C. Leahy R. M. (2001). Electromagnetic brain mapping. IEEE Signal. Process. Mag. 18, 14–30

[B2] Bell A. Sejnowski T. (1995). An information-maximization approach to blind separation and blind deconvolution. Neural Comput. 7, 1129–1159 7584893 10.1162/neco.1995.7.6.1129

[B3] Brankačk J. Stewart M. Fox S. E. (1993). Current source density analysis of the hippocampal theta rhythm: associated sustained potentials and candidate synaptic generators. Brain Res. 615, 310–327 8364740 10.1016/0006-8993(93)90043-m

[B4] Bullock T. H. McClune M. C. Enright J. T. (2003). Are the electroencephalograms mainly rhythmic? Assessment of periodicity in wide-band time series. Neuroscience 121, 233–252 10.1016/S0306-4522(03)00208-2 12946714

[B5] Burns S. P. Xing D. (2011). Is gamma-band activity in the local field potential of V1 cortex a “clock” or filtered noise? J. Neurosci. 31, 9658–9664 10.1523/JNEUROSCI.0660-11.2011 21715631 PMC3518456

[B6] Buzsáki G. Leung L. W. Vanderwolf C. H. (1983). Cellular bases of hippocampal EEG in the behaving rat. Brain Res. 287, 139–171 6357356 10.1016/0165-0173(83)90037-1

[B7] Buzsáki G. Wang X. J. (2012). Mechanisms of gamma oscillations. Annu. Rev. Neurosci. 35, 203–225 22443509 10.1146/annurev-neuro-062111-150444 PMC4049541

[B8] Chang M. H. Armstrong K. M. Moore T. (2012). Dissociation of response variability from firing rate effects in frontal eye field neurons during visual stimulation, working memory, and attention. J. Neurosci. 32, 2204–2216 10.1523/JNEUROSCI.2967-11.2012 22323732 PMC3439504

[B9] Choi S. Cichocki A. Park H. M. Lee S. Y. (2005). Blind source separation and independent component analysis: a review. Neural Inf. Process. Lett. Rev. 6, 1–57

[B10] Comon P. (1994). Independent component analysis. A new concept? Signal Process. 138 287–314

[B11] Csicsvari J. Jamieson B. Wise K. D. Buzsáki G. (2003). Mechanisms of gamma oscillations in the hippocampus of the behaving rat. Neuron 37, 311–322 12546825 10.1016/s0896-6273(02)01169-8

[B12] Czurkó A. Hirase H. Csicsvari J. Buzsáki G. (1999). Sustained activation of hippocampal pyramidal cells by ‘space clamping’ in a running wheel. Eur. J. Neurosci. 11, 344–352 10.1046/j.1460-9568.1999.00446.x 9987037

[B13] Dement W. Kleitman N. (1957). Cyclic variations in EEG during sleep and their relation to eye movements, body motility and dreaming. Electroencephalogr. Clin. Neurophysiol. 9, 673–690 13480240 10.1016/0013-4694(57)90088-3

[B14] Di S. Baumgartner C. Barth D. S. (1990). Laminar analysis of extracellular field potentials in rat vibrissa/barrel cortex. J. Neurophysiol. 63, 832–840 2341880 10.1152/jn.1990.63.4.832

[B15] Elul R. (1972). The genesis of the EEG. Int. Rev. Neurobiol. 15, 228–272 10.1016/s0074-7742(08)60333-5 4949975

[B16] Fernández-Ruiz A. Makarov V. A. Benito N. Herreras O. (2012a). Schaffer-specific local field potentials reflect discrete excitatory events at gamma-frequency that may fire postsynaptic hippocampal CA1 units. J. Neurosci. 32, 5165–5176 10.1523/JNEUROSCI.4499-11.2012 22496562 PMC6622107

[B17] Fernández-Ruiz A. Makarov V. A. Herreras O. (2012b). Sustained increase of spontaneous input and spike transfer in the CA3-CA1 pathway following long term potentiation *in vivo*. Front. Neural Circuits 6:71 10.3389/fncir.2012.00071 23060752 PMC3464490

[B18] Florian G. Andrew C. Pfurtscheller G. (1998). Do changes in coherence always reflect changes in functional coupling? Electroencephalogr. Clin. Neurophysiol. 106, 87–91 9680169 10.1016/s0013-4694(97)00105-3

[B19] Freeman J. A. Nicholson C. (1975). Experimental optimization of current source-density technique for anuran cerebellum. J. Neurophysiol. 38, 369–382 165272 10.1152/jn.1975.38.2.369

[B20] Fries P. Nikolic D. Singer W. (2007). The gamma cycle. Trends Neurosci. 30, 309–316 17555828 10.1016/j.tins.2007.05.005

[B21] Gloor P. (1985). Neuronal generators and the problem of localization in electroencephalography: application of volume conductor theory to electroencephalography. J. Clin. Neurophysiol. 2, 327–354 4056020 10.1097/00004691-198510000-00002

[B22] Gray C. M. König P. Engel A. K. Singer W. (1989). Oscillatory responses in cat visual cortex exhibit inter-columnar synchronization which reflects global stimulus properties. Nature 338, 334–337 10.1038/338334a0 2922061

[B23] Happel M. F. Jeschke M. Ohl F. W. (2010). Spectral integration in primary auditory cortex attributable to temporally precise convergence of thalamocortical and intracortical input. J. Neurosci. 30, 11114–11127 10.1523/JNEUROSCI.0689-10.2010 20720119 PMC6633479

[B24] Herreras O. (1990). Propagating dendritic action potential mediates synaptic transmission in CA1 pyramidal cells *in situ*. J. Neurophysiol. 64, 1429–1441 2178183 10.1152/jn.1990.64.5.1429

[B25] Jenkins G. M. Watts D. G. (1968). Spectral Analysis and its Applications. San Francisco, CA: Holden-Day

[B26] Kalcher K. Huf W. Boubela R. N. Filzmoser P. Pezawas L. Biswal B. (2012). Fully exploratory network independent component analysis of the 1000 functional connectomes database. Front. Hum. Neurosci. 6:301 10.3389/fnhum.2012.00301 23133413 PMC3490136

[B27] Kocsis B. Bragin A. Buzsáki G. (1999). Interdependence of multiple theta generators in the hippocampus: a partial coherence analysis. J. Neurosci. 19, 6200–6212 10407056 10.1523/JNEUROSCI.19-14-06200.1999 PMC6783086

[B28] Korovaichuk A. Makarova J. Makarov V. A. Benito N. Herreras O. (2010). Minor contribution of principal excitatory pathways to hippocampal LFPs in the anesthetized rat: a combined independent component and current source density study. J. Neurophysiol. 104, 484–497 10.1152/jn.00297.2010 20463202

[B29] Kuki T. Ohshiro T. Ito S. Ji Z. G. Fukazawa Y. Matsuzaka Y. (2012). Frequency-dependent entrainment of neocortical slow oscillation to repeated optogenetic stimulation in the anesthetized rat. Neurosci. Res. [Epub ahead of print]. 10.1016/j.neures.2012.10.007 23154073

[B30] Leung L. S. (1979). Potentials evoked by alvear tract in hippocampal CA 1 region in rats. II. Spatial field analysis. J. Neurophysiol. 42, 1571–1589 501390 10.1152/jn.1979.42.6.1571

[B31] López-Aguado L. Ibarz J. M. Herreras O. (2001). Activity-dependent changes of tissue resistivity in the CA1 region *in vivo* are layer-specific: modulation of evoked potentials. Neuroscience 108, 249–262 10.1016/S0306-4522(01)00417-1 11734358

[B32] López-Aguado L. Ibarz J. M. Varona P. Herreras O. (2002). Structural inhomogeneities differentially modulate action currents and population spikes initiated in the axon or dendrites. J. Neurophysiol. 88, 2809–2820 10.1152/jn.00183.2002 12424314

[B33] Lorente de No R. (1947). Analysis of the distribution of the action currents of nerve in volume conductors. Stud. Rockefeller Inst. Med. Res. Repr. 132, 384–477 20261890

[B34] Makarov V. A. Makarova J. Herreras O. (2010). Disentanglement of local field potential sources by independent component analysis. J. Comp. Neurosci. 29, 445–457 10.1007/s10827-009-0206-y 20094907

[B35] Makarova J. Ibarz J. M. Makarov V. A. Benito N. Herreras O. (2011). Parallel readout of pathway-specific inputs to laminated brain structures. Front. Syst. Neurosci. 5:77 10.3389/fnsys.2011.00077 21949504 PMC3171694

[B36] Makeig S. Jung T. P. Bell A. J. Ghahremani D. Sejnowski T. J. (1997). Blind separation of auditory event-related brain responses into independent components. Proc. Natl. Acad. Sci. U.S.A. 94, 10979–10984 9380745 10.1073/pnas.94.20.10979 PMC23551

[B37] Mann E. O. Paulsen O. (2007). Role of GABAergic inhibition in hippocampal network oscillations. Trends Neurosci. 30, 343–349 10.1016/j.tins.2007.05.003 17532059

[B38] Montgomery S. M. Betancur M. I. Buzsáki G. (2009). Behavior-dependent coordination of multiple theta dipoles in the hippocampus. J. Neurosci. 29, 1381–1394 10.1523/JNEUROSCI.4339-08.2009 19193885 PMC2768079

[B39] Nunez P. L. Srinivasan R. (2010). Scale and frequency chauvinism in brain dynamics: too much emphasis on γ band oscillations. Brain Struct. Funct. 215, 67–71 10.1007/s00429-010-0277-6 20890614 PMC2998274

[B40] Oppenheim A. V. Schafer R. W. (1989). Discrete-Time Signal Processing. Englewood Cliffs, NJ: Prentice Hall

[B41] Penttonen M. Kamondi A. Acsády L. Buzsáki G. (1998). Gamma frequency oscillation in the hippocampus of the rat: intracellular analysis *in vivo*. Eur. J. Neurosci. 10, 718–728 10.1046/j.1460-9568.1998.00096.x 9749733

[B42] Ray S. Maunsell J. H. R. (2010). Differences in gamma frequencies across visual cortex restrict their possible use in computation. Neuron 67, 885–896 10.1016/j.neuron.2010.08.004 20826318 PMC3001273

[B43] Ray S. Maunsell J. H. R. (2011). Network rhythms influence the relationship between spike-triggered local field potential and functional connectivity. J. Neurosci. 31, 12674–12682 10.1523/JNEUROSCI.1856-11.2011 21880928 PMC3488382

[B44] Reich D. S. Victor J. D. Knight B. W. Ozaki T. Kaplan E. (1997). Response variability and timing precision of neuronal spike trains *in vivo*. J. Neurophysiol. 77, 2836–2841 9163398 10.1152/jn.1997.77.5.2836

[B45] Rivas J. Gaztelu J. M. García-Austt E. (1996). Changes in hippocampal cell discharge patterns and theta rhythm spectral properties as a function of walking velocity in the guinea pig. Exp. Brain Res. 108, 113–118 8721159 10.1007/BF00242908

[B46] Schmidt R. Diba K. Leibold C. Schmitz D. Buzsáki G. Kempter R. (2009). Single-trial phase precession in the hippocampus. J. Neurosci. 29, 13232–13241 10.1523/JNEUROSCI.2270-09.2009 19846711 PMC2830422

[B47] Schroeder C. E. Mehta A. D. Givre S. J. (1998). A spatiotemporal profile of visual system activation revealed by current source density analysis in the awake macaque. Cereb. Cortex 8, 575–592 9823479 10.1093/cercor/8.7.575

[B48] Singer W. Gray C. M. (1995). Visual feature integration and the temporal correlation hypothesis. Annu. Rev. Neurosci. 18, 555–586 10.1146/annurev.ne.18.030195.003011 7605074

[B49] Srinivasan R. Willian R. W. Nunez P. L. (2006). Source analysis of EEG oscillations using high-resolution EEG and MEG. Prog. Brain Res. 159, 29–42 10.1016/S0079-6123(06)59003-X 17071222 PMC1995013

[B50] Tang A. C. Pearlmutter B. A. Malaszenko N. A. Phung D. B. (2002). Independent components of magnetoencephalography: single-trial response onset time. Neuroimage 17, 1773–1789 10.1006/nimg.2002.1320 12498751

[B51] Varona P. Ibarz J. M. López-Aguado L. Herreras O. (2000). Macroscopic and subcellular factors shaping CA1 population spikes. J. Neurophysiol. 83, 2192–2208 10758128 10.1152/jn.2000.83.4.2192

[B52] Wu K. Canning K. J. Leung L. S. (1998). Functional interconnections between CA3 and the dentate gyrus revealed by current source density analysis. Hippocampus 8, 217–230 10.1002/(SICI)1098-1063(1998)8:3<217::AID-HIPO5>3.0.CO;2-G 9662137

[B53] Ylinen A. Bragin A. Nádasdy Z. Jandó G. Szabó I. Sik A. (1995). Sharp wave-associated high-frequency oscillation (200 Hz) in the intact hippocampus: network and intracellular mechanisms. J. Neurosci. 15, 30–46 7823136 10.1523/JNEUROSCI.15-01-00030.1995 PMC6578299

